# Modulation of associations between education years and cortical volume in Alzheimer’s disease vulnerable brain regions by Aβ deposition and *APOE* ε4 carrier status in cognitively normal older adults

**DOI:** 10.3389/fnagi.2023.1248531

**Published:** 2023-09-27

**Authors:** Hak-Bin Kim, Sung-Hwan Kim, Yoo Hyun Um, Sheng-Min Wang, Regina E. Y. Kim, Yeong Sim Choe, Jiyeon Lee, Donghyeon Kim, Hyun Kook Lim, Chang Uk Lee, Dong Woo Kang

**Affiliations:** ^1^Department of Psychiatry, Seoul St. Mary’s Hospital, College of Medicine, The Catholic University of Korea, Seoul, Republic of Korea; ^2^Department of Psychiatry, Yeouido St. Mary’s Hospital, College of Medicine, The Catholic University of Korea, Seoul, Republic of Korea; ^3^Department of Psychiatry, St. Vincent’s Hospital, College of Medicine, The Catholic University of Korea, Suwon, Republic of Korea; ^4^Research Institute, NEUROPHET Inc., Seoul, Republic of Korea

**Keywords:** amyloid-beta, *APOE* ε4 allele, cortical volume, cognitive function, cognitively normal older adults

## Abstract

**Background:**

Education years, as a measure of cognitive reserve, have been shown to affect the progression of Alzheimer’s disease (AD), both pathologically and clinically. However, inconsistent results have been reported regarding the association between years of education and intermediate structural changes in AD-vulnerable brain regions, particularly when AD risk factors were not considered during the preclinical phase.

**Objective:**

This study aimed to examine how Aβ deposition and *APOE* ε4 carrier status moderate the relationship between years of education and cortical volume in AD-vulnerable regions among cognitively normal older adults.

**Methods:**

A total of 121 participants underwent structural MRI, [^18^F] flutemetamol PET-CT imaging, and neuropsychological battery assessment. Multiple regression analysis was conducted to examine the interaction between years of education and the effects of potential modifiers on cortical volume. The associations between cortical volume and neuropsychological performance were further explored in subgroups categorized based on AD risk factors.

**Results:**

The cortical volume of the left lateral occipital cortex and bilateral fusiform gyrus demonstrated a significant differential association with years of education, depending on the presence of Aβ deposition and *APOE* ε4 carrier status. Furthermore, a significant relationship between the cortical volume of the bilateral fusiform gyrus and AD-nonspecific cognitive function was predominantly observed in individuals without AD risk factors.

**Conclusion:**

AD risk factors exerted varying influences on the association between years of education and cortical volume during the preclinical phase. Further investigations into the long-term implications of these findings would enhance our understanding of cognitive reserves in the preclinical stages of AD.

## Introduction

Alzheimer’s disease (AD) is a neurodegenerative disease characterized by the deposition of amyloid-beta (Aβ) and tau, which leads to deteriorating changes in the brain and impairment of cognitive function and the ability to perform daily activities (Bloom, 2014). In addition, the *APOE* ε4 allele modulates the penetrance and weight of the Aβ pathophysiological cascade (Frisoni et al., 2022) and is responsible for the largest proportion of genetic risk factors for sporadic AD (Sims et al., 2020). Additionally, the *APOE* ε4 allele increases the risk of sporadic AD in a dose-dependent manner (Corder et al., 1993). It has also been reported to affect the deposition (DeMattos et al., 2004), clearance of Aβ (Castellano et al., 2011), and modulation of tau binding (Small et al., 2009).

The clinical symptoms of AD often do not match the degree of neuropathological changes observed (Katzman et al., 1988), which are attributed to an individual’s cognitive reserve, brain reserve and maintenance (Stern et al., 2020). Among these factors, cognitive reserve is the ability to tolerate brain damage or neuropathological changes in order to maintain cognitive and functional status through the use of compensatory neural mechanisms (Stern et al., 2020). Crucially, cognitive reserve influences when dementia begins, how rapidly cognitive decline progresses, and the extent of the pathology that results in dementia (Stern, 2012). Individuals with high cognitive reserve might experience a delay in the onset of dementia (Valenzuela and Sachdev, 2006b), but once symptoms begin, they may progress faster due to a larger load of latent pathology (van Loenhoud et al., 2019).

Convenience proxies related to various socio-behavioral factors that contribute to the formation of cognitive reserve are used, among which years of education is frequently used (Stern et al., 2020). This is because educational attainment is standardized and easy to measure. Various studies have shown interesting correlations between education level and AD. Among older adults and dementia patients with comparable degrees of cognitive decline, individuals with higher education levels tend to show more advanced Aβ pathology (Roe et al., 2008). Furthermore, longer education periods appear to offer a protective effect against cognitive decline in elderly individuals (Valenzuela and Sachdev, 2006a).

Brain structural parameters, which are both reliable and stable, provide another way of tracking AD progression (Fox and Freeborough, 1997; Bobinski et al., 1999; Whitwell et al., 2012). A previous post-mortem study showed that individuals with higher education levels had greater brain weights than those with fewer years of education (Brayne et al., 2010). However, another study contradicted this finding, indicating a negative relationship between years of education and cortical thickness (Querbes et al., 2009).

Despite several attempts to establish a correlation between years of education and cortical atrophy, results have been inconclusive. Some studies have found increased cortical atrophy (as indicated by increased sulcal CSF volume) with more years of education (Coffey et al., 1999), while others have reported no such relationship (Christensen et al., 2009; Seo et al., 2011). Even within the cognitively normal group, the data are mixed, with some studies suggesting increased cortical thickness in certain brain regions with more years of education (Foubert-Samier et al., 2012; Liu et al., 2012).

These inconsistencies in the relationship between years of education and cortical atrophy could be due to the lack of consideration of other contributing risk factors such as Aβ deposition, *APOE* ε4 carrier status, and tauopathy, which can all influence cortical atrophy (Harrison et al., 2019; Ossenkoppele et al., 2019). In addition, there is a dearth of research examining the association between years of education and cortical atrophy during preclinical stages of AD. Therefore, future research into this association during the early stages of AD would significantly enrich our understanding of the role that cognitive reserves play in the progression of AD.

In this regard, we aimed to evaluate how Aβ deposition and *APOE* ε4 carrier status moderate the relationship between years of education and cortical volume in AD-vulnerable regions among cognitively normal older adults. In addition, we assessed whether the progression of Aβ deposition, particularly in the presence of positive Aβ deposition and the *APOE* ε4 allele, elucidates the mechanism by which years of education affect cortical volume.

For regions of interest in the cortical volume that show significant education years-by-AD risk factor interaction, we also set out to confirm whether the association with cognitive function varied depending on the AD risk factors. Additionally, we evaluated cortical atrophy not only in brain regions where Aβ deposition is mainly found but also in tauopathy-vulnerable regions, considering that tauopathy was not directly evaluated in this study.

## Materials and methods

### Participants

One hundred twenty-one subjects aged 55–85 years were recruited from volunteers registered in the Catholic Aging Brain Imaging database, which contains brain scans of patients who visited the outpatient clinic at the Catholic Brain Health Center, Yeouido St. Mary’s Hospital, The Catholic University of Korea, from 2018 to 2020.

Cognitive function of all subjects was assessed using the Korean version of the Consortium to Establish a Registry for AD (CERAD-K) (Lee et al., 2002). The measurements included assessments of the Korean version of the verbal fluency (VF) test, 15-item Boston Naming Test, Mini-Mental State Examination (MMSE-K) (Park, 1989), word list memory (WLM), word list recall (WLR), word list recognition (WLRc), constructional praxis (CP), and constructional recall (CR). In addition, the total memory (TM) scores were obtained by summing the scores from the WLM, WLR, and WLRc tests. The total CERAD-K score was calculated by summing all subcategory scores, excluding the MMSE-K scores. Higher Trail Making Test B scores indicated lower executive function. Details regarding the use of specific tests and the review process are described in the Supplementary material.

The inclusion criteria were as follows: (1) unimpaired delayed memory function, quantified by scoring above age-, sex-, and education-adjusted cutoffs on the WLR domains; (2) MMSE-K score between 24 and 30; (3) Clinical Dementia Rating score of 0; (4) Memory Box score of 0; (5) normal cognitive function based on the absence of significant impairment in cognitive function or activities of daily living; and (6) no family history of AD. We excluded participants with a history of alcoholism, drug abuse, head trauma, or psychiatric disorders, those taking any psychotropic medications (e.g., cholinesterase inhibitors, antidepressants, benzodiazepines, and antipsychotics) (*N* = 37), those with uncontrolled multiple cardiovascular risk factors (e.g., uncontrolled arterial hypertension, diabetes mellitus, dyslipidemia, cardiac disease including coronary heart disease, arrhythmia (*N* = 52), etc.), and those with evidence of subcortical ischemic changes corresponding to a score ≥ 2 on the Fazeka scale (*N* = 24) (Fazekas et al., 1987). Specific criteria for multiple uncontrolled cardiovascular risk factors are detailed in the Supplementary material.

T2-weighted fluid-attenuated inversion recovery (FLAIR) data were acquired to exclude vascular lesions or other diseases objectively. Participants underwent [^18^F] flutemetamol (FMM) PET-CT within 3 months of magnetic resonance imaging (MRI).

The procedures for *APOE* genotyping are described in Supplementary material. Considering the protective effects of the *APOE* ε2 allele (Li et al., 2020), we excluded participants with the *APOE* ε2 allele (*N* = 38). If a participant had at least one *APOE* ε4 allele, they were categorized as *APOE* ε4 carriers; if they had no *APOE* ε4 allele, they were categorized as *APOE* ε4 non-carriers. This study was conducted under the ethical and safety guidelines set forth by the Institutional Review Board of the Catholic University of Korea, which approved all research activities (SC18TESI0143). Written informed consent was obtained from all the participants.

### Structural MRI data acquisition

Imaging data were collected by the Department of Radiology of Yeouido Saint Mary’s Hospital at the Catholic University of Korea using a 3-T Siemens Skyra MRI machine and 32-channel Siemens head coil (Siemens Medical Solutions, Erlangen, Germany). The scanning parameters of the T1-weighted three-dimensional magnetization-prepared rapid gradient echo sequences were as follows; echo time (TE) = 2.6 ms, repetition time (TR) = 1,940 ms, inversion time (TI) = 979 ms, flip angle (FA) = 9o, field of view = 250 × 250 mm, matrix = 256 × 256, and voxel size = 1.0 × 1.0 × 1.0 mm^3^. The FLAIR MRI sequences were as follows: TE = 135 ms; TR = 9,000 ms; TI = 2,200 ms; FA = 90o; FOV = 220×220 mm; matrix = 356 × 231; and voxel size = 1.0 × 1.0 × 1.0 mm^3^.

### Structural MRI processing

All T1-weighted MRI scans were processed using a previously proposed method based on deep-learning-based segmentation for structural volume measurements (Lee et al., 2020). AQUA from Neurophet Inc. was used for T1 MRI processing, where the U-NET++ deep learning architecture was utilized. The method used in this study showed high dice similarity coefficients overlap with experts and high reliability across multicenter studies (Lee et al., 2020). Furthermore, the method used in this study, first proposed by Lee et al. (2020), was validated using a multirace dataset (Kim et al., 2020). This deep learning-based segmentation method was explicitly compared with classical tools such as FreeSurfer, demonstrating superior reliability for a multicenter study dataset. The advantage of using this method lies in its enhanced performance across diverse populations, as it was intensively tested using the East Asian dataset, highlighting significant improvements over classical tools where differences between the Caucasian and East Asian populations have been known in previous studies (Tang et al., 2018; Kang et al., 2020). Once the ROIs were automatically delineated, the subregions were merged into the cortical brain volume for analysis.

### Cortical volume procedure

The mean cortical volumes of the areas of interest were extracted from each participant based on deep learning-based segmentation. We focused on the cortical Braak regions, which are typically affected in AD (Braak and Braak, 1997), with the aim of the entorhinal cortex, hippocampus, fusiform gyrus, superior, middle, and inferior temporal gyrus, insula, temporal pole, posterior cingulate cortex, precuneus, and lateral occipital cortex. The segmentation procedure, also performed as implemented in AQUA, enabled us to obtain a measure of the estimated intracranial volume (Lee et al., 2020), which is based on the scale factor used for atlas registration.

### [^18^F]-flutemetamol PET image acquisition and processing

FMM-PET data were collected and analyzed as described previously (Thurfjell et al., 2014). Static PET scans were acquired 90–110 min after 185 MBq of FMM injection. MRI for each participant was used to co-register and define the ROIs and correct partial volume effects that arose from the expansion of the cerebrospinal spaces accompanying cerebral atrophy using a geometric transfer matrix.

### SUVR calculation

Semi-quantification of FMM uptake on PET/CT was performed by obtaining the standardized uptake value ratios (SUVRs). SUVRs were measured using SCALE PET from Neurophet Inc. (Seoul, Republic of Korea) (Lee et al., 2022). T1 MRI was used along with PET imaging for structural information. The volumes of interest (VOIs) were restricted to gray matter, covering the frontal, superior parietal, lateral temporal, anterior, and posterior cingulate cortex/precuneus regions. These VOIs were also considered in a previous study (Thurfjell et al., 2014). The reference region for the SUVR calculations is the pons. The mean uptake counts of each VOIs and reference region were measured on the preprocessed image. The regional SUVR was calculated as the ratio of each cortical regional mean count to the pons mean count (SUVR_PONS_). The global cortical average (composite SUVR) was calculated by averaging the regional cortical SUVRs weighted by size. A detailed description was provided in our previous study (Lee et al., 2022). We used a cut-off of 0.62 for “positive (Aβ+)” versus ‘negative (Aβ−)’ neocortical SUVR, consistent with the cut-off values used in a previous FMM PET study (Thurfjell et al., 2014). PET scans classified as negative for Aβ accumulation also exhibited normal visual readings.

### Statistical analysis

Statistical analyses were performed using the R software (version 4.0.5), Jamovi (version 1.6.23),^1^ and SPM 12. Assumptions of normality were tested for continuous variables using the Kolmogorov–Smirnov test in the R software; all data demonstrated a normal distribution. The two-sample *t*-test and chi-square (χ^2^) tests were used to probe for differences in demographic variables, clinical data, global FMM SUVR_PONS_, and neuropsychological performance scores between the Aβ + and Aβ- groups. All statistical analyses were conducted considering a two-tailed *p*-value <0.05 to define statistical significance.

For the cortical volume in AD vulnerable regions, we performed partial correlation analysis to evaluate the association with years of education, adjusting for age, sex, total intracranial volume, Aβ deposition, and *APOE* ε4 carrier status. In addition, multiple regression analysis was performed to evaluate the education years-by-effect modifiers (Aβ deposition and *APOE* ε4 carrier status) interaction, adjusting for age, sex, total intracranial volume, and effect modifiers not included in each interaction evaluation. Additionally, we evaluated the interaction between Aβ deposition and *APOE* ε4 carrier status for cortical volume in AD-vulnerable regions displaying the education years-by-effect modifier interaction to clarify the modulating effect of each AD risk factor.

To examine whether global FMM SUVR_PONS_ mediated the association between years of education and cortical volume in regions of interest in each Aβ + group and *APOE* ε4 carriers, mediation analysis was performed. The data were analyzed using Hayes’ PROCESS macro model 4 for SPSS (version 26.0) to investigate the potential mediation effect. This model was chosen because it enables testing of the indirect effect of the independent variable (X) on the dependent variable (Y) through the mediator (M). In our model, years of education was the independent variable (X), cortical volume in the region of interest was the dependent variable (Y), and global FMM SUVR_PONS_ was the mediating variable (M). The total effect of X on Y, the direct effect of X on Y controlling for M, and the indirect effect of X on Y through M were analyzed. A 1000-sample bootstrapping was used to generate upper and lower limits for confidence intervals for indirect effects to minimize distributional assumptions in smaller sample sizes (Preacher and Hayes, 2004). When the 95% confidence interval did not include 0, the indirect effect was considered significant (*α* = 0.05).

For cortical volume in AD susceptible regions showing the interaction of years of education by effect modifier, we evaluated whether the differential association with neuropsychological performance exists by AD risk factors, controlling for age, sex, years of education, total intracranial volume, and effect modifier not included in each analysis. Moreover, we examined the association with the neuropsychological performance scores in each group categorized by the effect modifier (Aβ + vs. Aβ- group; *APOE* ε4 carrier vs. *APOE* ε4 non-carrier) using multiple regression analysis, adjusting for age, sex, years of education, total intracranial volume, and effect modifier not included in each analysis. All statistical analyses used a two-tailed *p*-value <0.05 to define statistical significance.

## Results

### Baseline demographic and clinical data

Table 1A shows the baseline demographic data for the Aβ + and Aβ − groups of cognitively normal older adults. The mean age of the Aβ − group was 68.7 years, while that of the Aβ + group was 70.1 years. In both groups, females were overrepresented compared to males (68.7 and 62.3%, respectively). The mean number of years of education was 13.7 years in the Aβ − and 13.9 years in the Aβ + group. There were no significant differences in age, age distribution, sex, and number of years of education between the Aβ + and Aβ − groups. In addition, the proportion of *APOE* ε4 carriers was 41.9% in the Aβ + group, which was significantly higher than the 20% in the Aβ − group. The Global FMM SUVR_PONS_ value, which was used to categorize the Aβ + and Aβ − groups, was also significantly higher in the Aβ + group. No significant differences were found in neuropsychological performance scores between the Aβ + and Aβ − groups.

**Table 1:** Demographic and clinical characteristics of cognitively normal older adults.

(A) Cognitively normal older adults stratified by Aβ deposition			
	CN (Aβ−) (*n* = 90)	CN (Aβ +) (*n* = 31)	*p*-value
Age (years)	68.7 ± 7.3	70.1 ± 6.8	0.371
55–59 (%)	9 (10%)	3 (9.7%)	0.502
60–69 (%)	44 (48.9%)	11 (35.5%)	
70–79 (%)	31 (34.4%)	13 (41.9%)	
80–85 (%)	6 (6.7%)	4 (12.9%)	
Sex (Female, %)	61 (67.8%)	19 (61.3%)	0.661
Education years	13.7 ± 3.1	13.9 ± 2.6	0.799
*APOE* ε4 carrier status (carrier, %)	18 (20.0%)	13 (41.9%)	0.03
Global SUVR_PONS_	0.55 ± 0.03	0.74 ± 0.24	< 0.001
MMSE-K	28.2 ± 1.4	28.2 ± 1.6	0.950
CERAD-K VF	16.0 ± 4.3	16.4 ± 4.1	0.653
CERAD-K BNT	12.8 ± 1.7	12.6 ± 1.8	0.716
CERAD-K WLM	20.0 ± 3.3	19.4 ± 3.6	0.419
CERAD-K CP	10.8 ± 0.6	10.9 ± 0.3	0.756
CERAD-K WLR	6.8 ± 1.6	6.5 ± 1.8	0.533
CERAD-K WLRc	9.5 ± 0.8	9.3 ± 1.0	0.161
CERAD-K CR	7.9 ± 2.8	7.8 ± 2.7	0.796
CERAD-K TM	36.3 ± 4.9	35.2 ± 5.7	0.331
CERAD-K Total	83.8 ± 10.2	82.9 ± 12.0	0.688

(B) Cognitively normal older adults stratified by *APOE* ε4 carrier status			
	*APOE* ε4 non-carrier (*n* = 90)	*APOE* ε4 carrier (*n* = 31)	*p* value
Age (years)	69.1 ± 7.2	68.8 ± 7.2	0.839
55–59 (%)	8 (8.9%)	4 (12.9%)	0.726
60–69 (%)	43 (47.8%)	12 (38.7%)	
70–79 (%)	31 (34.4%)	13 (41.9%)	
80–85 (%)	8 (8.9%)	2 (6.5%)	
Sex (Female, %)	59 (65.6%)	21 (67.7%)	0.999
Education years	13.7 ± 3.2	13.8 ± 2.5	0.908
Positivity of Aβ deposition (%)	18 (20.0%)	13 (41.9%)	0.03
Global SUVR_PONS_	0.60 ± 0.17	0.60 ± 0.08	0.961
MMSE-K	28.3 ± 1.5	28.2 ± 1.3	0.919
CERAD-K VF	16.3 ± 4.4	15.8 ± 3.7	0.589
CERAD-K BNT	12.9 ± 1.7	12.2 ± 1.8	0.062
CERAD-K WLM	19.8 ± 3.5	20.1 ± 3.1	0.623
CERAD-K CP	10.8 ± 0.6	10.9 ± 0.2	0.093
CERAD-K WLR	6.7 ± 1.7	6.7 ± 1.5	0.966
CERAD-K WLRc	9.5 ± 0.8	9.4 ± 1.1	0.484
CERAD-K CR	7.9 ± 2.8	7.9 ± 2.7	0.988
CERAD-K TM	35.9 ± 5.3	36.2 ± 4.8	0.841
CERAD-K Total	75.9 ± 9.1	75.1 ± 8.3	0.662

Data are presented as the mean ± SD unless indicated otherwise. MMSE-K, The Korean version of Mini Mental Status Examination; CERAD-K, Korean version of Consortium to Establish a Registry for Alzheimer’s Disease; VF, Verbal Fluency; BNT, 15-item Boston Naming Test; MMSE, Mini Mental Status Examination; WLM, Word List Memory; CP, Constructional Praxis; WLR, Word List Recall; WLRc, Word List Recognition; CR, Constructional Recall; TM, sum of the respective scores from the WLM, WLR, and WLRc; Total, sum of all subcategory scores, excluding the MMSE-K scores; Aβ–, negative amyloid beta deposition; Aβ+, positive amyloid beta deposition. SUVR_PONS_, standardized uptake value ratios of [^18^F] flutemetamol, using pons as reference region.

Baseline demographic information for older adults with normal cognitive function, who are *APOE* ε4 carriers and non-carriers, is presented in Table 1B. The *APOE* ε4 non-carriers had an average age of 69.1 years, compared to 68.8 years for the *APOE* ε4 carriers. Female were more prevalent in both groups, comprising 65.6 and 67.7%, respectively. The mean education years was 13.7 years for *APOE* ε4 non-carriers and 13.8 years for *APOE* ε4 carriers. There were no notable differences between the two groups in terms of age, age distribution, sex, or education years. Additionally, the rate of Aβ positivity was significantly higher in the *APOE* ε4 carriers at 41.9%, compared to 20% in the non-carriers. However, there were no significant disparities in the global FMM SUVR_PONS_ value or neuropsychological performance scores between the *APOE* ε4 carriers and non-carriers.

### Association between education years and cortical volume according to Aβ deposition and *APOE* ε4 carrier status

**Figure 1:**
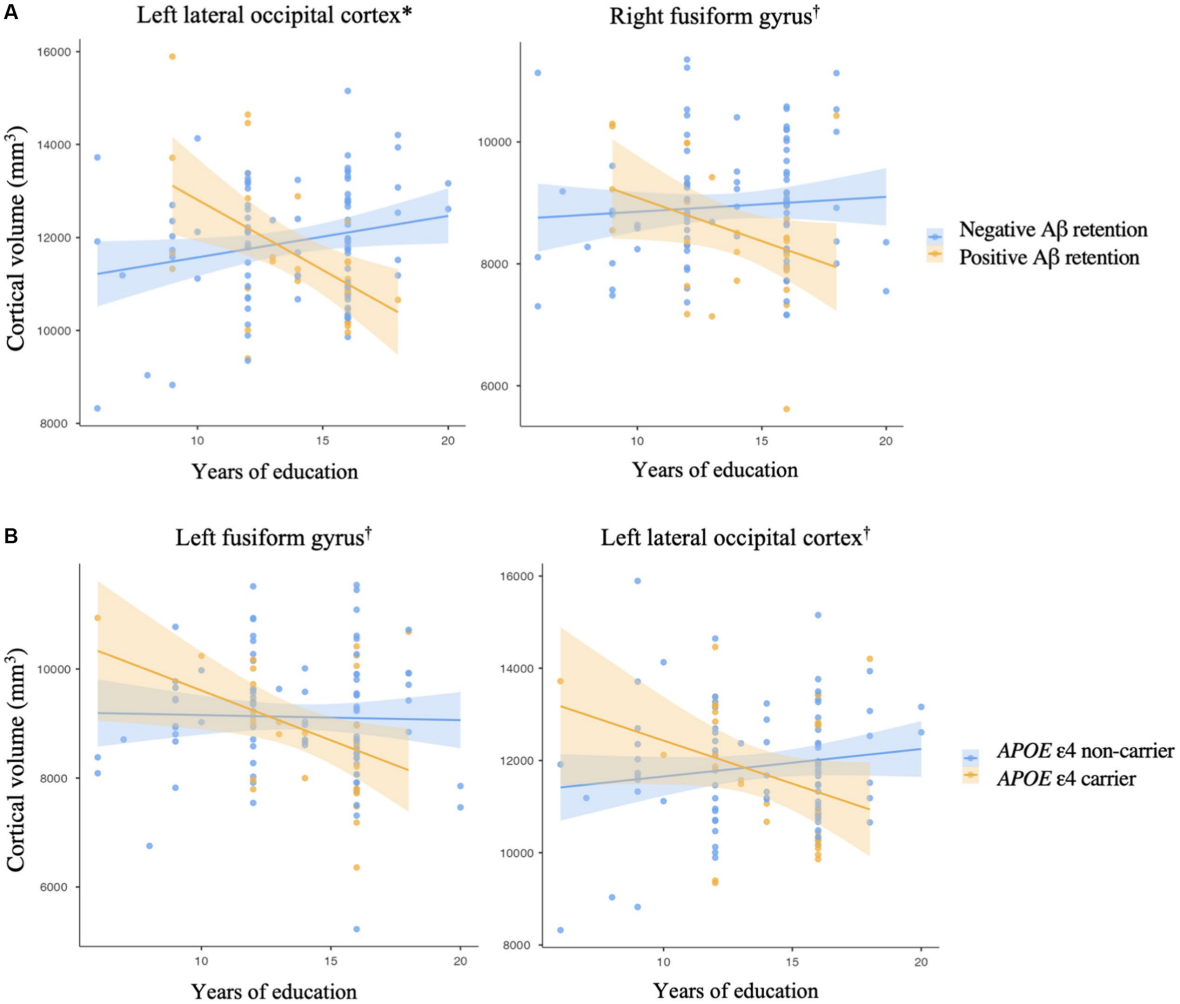
Impact of an interaction **(A)** between an education year and amyloid-beta retention on a cortical volume, **(B)** between an education year and *APOE* ε4 allele on a cortical volume. Brain areas are regions of interest. *p < 0.001, ^†^*p* < 0.05, by multiple regression analysis, **(A)** adjusted for the effects of age, sex, *APOE* ε4 carrier status, and total intracranial volume, **(B)** adjusted for the effects of age, sex, Aβ retention, and total intracranial volume. The shaded region surrounding the regression line indicates the standard error of the estimates.

There was no significant association between years of education and any cortical volumes in the regions of interest in cognitively normal older adults (Supplementary material). Among the regions of interest, the cortical volumes of the left lateral occipital cortex and right fusiform gyrus showed a significant differential association with years of education according to Aβ deposit positivity (Figure 1A and Table 2A, left lateral occipital cortex, *p* < 0.001; Table 2B, right fusiform gyrus, *p* = 0.049). These results can be attributed to the Aβ + group showing higher education years with lower cortical volume in cognitively normal older adults. However, there was no significant interaction between Aβ deposition and *APOE* ε4 carrier status for the volume of the left lateral occipital cortex (standardized β = 0.036, *p* = 0.935) and right fusiform gyrus (standardized β = 0.118, *p* = 0.791).

**Table 2:** Interaction between years of education and Alzheimer’s disease risk factors for cortical volume in regions of interest.

(A) Regions of interest: left lateral occipital cortex							
Fitted model	Estimate	SE	*t*	*p*	Stand. estimate	95% Confidence interval	
						Lower	Upper
Education years × *APOE* ε4 carrier status	−230.218	102.133	−2.254	0.025	−0.515	−0.967	−0.062
Education years × Aβ deposition	−474.114	108.9	−4.353	< 0.001	−1.060	−1.5419	−0.577

(B) Regions of interest: right fusiform gyrus							
Fitted model	Estimate	SE	*t*	*p*	Stand. estimate	95% Confidence interval	
						Lower	Upper
Education years × Aβ deposition	−171.190	82.7	−2.07	0.049	−0.499	−0.9763	−0.0215

(C) Regions of interest: left fusiform gyrus							
Fitted model	Estimate	SE	*t*	*p*	Stand. estimate	95% Confidence interval	
						Lower	Upper
Education years × *APOE* ε4 carrier status	−168.770	78.9	−2.168	0.028	−0.456	−0.874	−0.039

Regression models were adjusted for age, sex, total intracranial volume, and effect modifiers that were not included in each interaction evaluation. Estimate, unstandardized beta coefficients; Stand. Estimate, standardized beta coefficients; SE, standard error.

Additionally, the cortical volume of the left fusiform gyrus and left lateral occipital cortex showed a significantly distinctive association with years of education according to the *APOE* ε4 carrier status (Figure 1B and Table 2C, left fusiform gyrus, *p* = 0.028; Table 2A, left lateral occipital cortex, *p* = 0.025). However, there was no significant interaction between Aβ deposition and *APOE* ε4 carrier status for the volume of the left fusiform gyrus (standardized *β* = −0.333, *p* = 0.446) and left lateral occipital cortex (standardized *β* = 0.036, *p* = 0.935).

Table 3 shows the results of mediation analysis with years of education as an independent factor and cortical volume, displaying the education years-by-effect modifier interaction, as dependent factors in each Aβ + group (Tables 3A,B) and *APOE* ε4 carriers (Tables 3C,D). The proposed mediator was the global FMM SUVR_PONS_. In the Aβ + group, although there were significant direct and total effects of years of education on the cortical volume of the left lateral occipital cortex (direct effect, *β* = −0.5465, *p* = 0.003; total effect, *β* = −0.5157, *p* < 0.001, Table 3A), the global FMM SUVR_PONS_ did not mediate this effect (*β* = 0.0308, *p* = 0.535, Table 3A). Additionally, there was a total effect of years of education on the cortical volume of the right fusiform gyrus in the Aβ + group (*β* = −0.3427, *p* = 0.046, Table 2B). However, the global FMM SUVR_PONS_ did not have an indirect effect (*β* = 0.0108, *p* = 0.691, Table 3B). In the *APOE* ε4 carriers, there were no significant indirect, direct, or total effects of years of education on the cortical volume of the left fusiform gyrus (Table 3C). Additionally, although there were significant direct and total effects of years of education on cortical volume of the left lateral occipital cortex in *APOE* ε4 carriers (direct effect, *β* = −0.3890, *p* = 0.043; total effect, *β* = −0.4049, *p* = 0.015, Table 3D), the global FMM SUVR_PONS_ did not mediate this effect (*β* = −0.0159, *p* = 0.748, Table 3D).

**Table 3:** Mediation analysis between education years, Amyloid-β deposition, and cortical volume in cognitively normal older adults with **(A,B)** positive amyloid-β accumulation and **(C,D)** *APOE* ε4 allele: indirect and total effects.

(A) Regions of interest: left lateral occipital cortex								
Type	Effect	Estimate	SE	95% Confidence interval		β	z	p
				Lower	Upper			
Indirect	Education years ⇒ Aβ deposition⇒ Cortical volume	18.043	29.063	−13.891	101.207	0.031	0.621	0.535
Component	Education years ⇒ Aβ deposition	−0.012	0.016	−0.054	0.012	−0.130	−0.750	0.453
	Aβ deposition ⇒ Cortical volume	−1495.861	2024.994	−3501.624	4508.942	−0.238	−0.739	0.460
Direct	Education years ⇒ Cortical volume	−320.148	108.807	−555.653	−122.081	−0.547	−2.942	0.003
Total	Education years ⇒ Cortical volume	−302.105	91.647	−481.730	−122.480	−0.516	−3.296	< 0.001

(B) Regions of interest: right fusiform gyrus								
Type	Effect	Estimate	SE	95% Confidence interval		β	*z*	*p*
				Lower	Upper			
Indirect	Education years ⇒ Aβ deposition⇒ Cortical volume	4.503	11.3237	−12.610	32.391	0.011	0.398	0.691
Component	Education years ⇒ Aβ deposition	−0.012	0.0160	−0.054	0.011	−0.130	−0.753	0.451
	Aβ deposition ⇒ Cortical volume	−373.331	1238.372	−3135.8999	2754.321	−0.084	−0.301	0.763
Direct	Education years ⇒ Cortical volume	−147.077	82.967	−312.646	29.933	−0.354	−1.773	0.076
Total	Education years ⇒ Cortical volume	−142.574	71.360	−282.437	−2.711	−0.343	−1.998	0.046

(C) Regions of interest: left fusiform gyrus								
Type	Effect	Estimate	SE	95% Confidence interval		β	*z*	*p*
				Lower	Upper			
Indirect	Education years ⇒ Aβ deposition ⇒ Cortical volume	−1.172	18.093	−33.937	50.045	−0.002	−0.065	0.948
Component	Education years ⇒ Aβ deposition	0.002	0.005	−0.009	0.011	0.0590	0.368	0.713
	Aβ deposition ⇒ Cortical volume	−656.120	3922.324	−8436.272	7414.119	−0.034	−0.167	0.867
Direct	Education years ⇒ Cortical volume	−185.513	120.995	−403.564	70.483	−0.320	−1.533	0.125
Total	Education years ⇒ Cortical volume	−186.686	100.366	−383.400	10.028	−0.322	−1.860	0.063

(D) Regions of interest: left lateral occipital cortex								
Type	Effect	Estimate	SE	95% Confidence interval		β	*z*	*p*
				Lower	Upper			
Indirect	Education years ⇒ Aβ deposition ⇒ Cortical volume	−7.177	22.330	−42.888	54.075	−0.0160	−0.321	0.748
Component	Education years ⇒ Aβ deposition	0.002	0.005	−0.010	0.011	0.059	0.348	0.728
	Aβ deposition ⇒ Cortical volume	−4017.319	2582.954	−9326.432	932.822	−0.270	−1.555	0.120
Direct	Education years ⇒ Cortical volume	−175.459	86.860	−324.296	20.054	−0.389	−2.020	0.043
Total	Education years ⇒ Cortical volume	−182.636	75.300	−330.221	−35.050	−0.405	−2.425	0.015

Mediation analysis with education years (predictor, X), mediated by [^18^F] flutemetamol SUVR_PONS_ (mediator, M), predicting cortical volume in brain regions vulnerable to Alzheimer’s disease. Confidence intervals computed with method: Bootstrap percentiles. Betas are completely standardized effect sizes.

### Relationship between neuropsychological performance scores and cortical volume in regions of interest

Among the AD vulnerable regions showing the interaction of years of education by effect modifier, left fusiform gyrus cortical volume showed a significant interaction with Aβ deposition on CERAD-K WLRc score after adjusting for age, sex, years of education, total intracranial volume, and *APOE* ε4 carrier status (standardized *β* = 0.583, *p* = 0.021). This interaction contributed to a lower CERAD-K WLRc score in the Aβ + group, which had a lower cortical volume of the left fusiform gyrus. However, cortical volumes of other regions of interest did not show a significant interaction with AD risk factor for any of the neuropsychological test scores.

**Figure 2:**
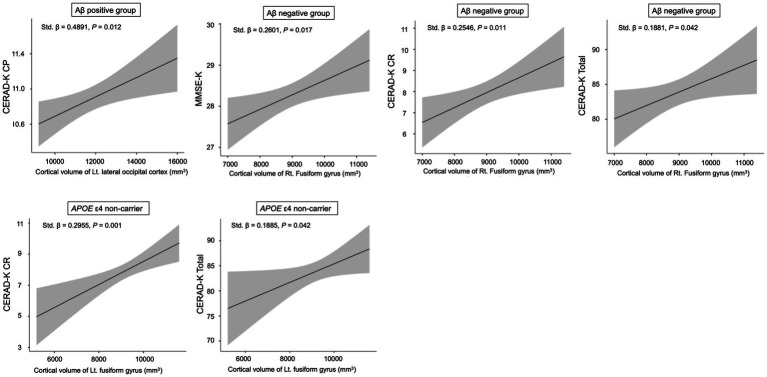
Relationship between neuropsychological performance scores and cortical volume in sub-categorized groups. Multiple regression analysis was adjusted for age, sex, years of education, total intracranial volume, and *APOE* ε4 carrier status (Aβ positive and negative groups) or Aβ deposition (*APOE* ε4 non-carrier). CP, constructional praxis; MMSE, mini mental status examination; CR, Constructional Recall; Total, sum of all subcategory scores, excluding the MMSE-K scores. The shaded region surrounding the regression line illustrates the standard error of the estimate.

For the cortical volume of the left lateral occipital cortex, there was a significant association with the CERAD-K CP score in the Aβ + group after adjusting for age, sex, years of education, total intracranial volume, and *APOE* ε4 carrier status (Figure 2, standardized *β* = 0.4891, *p* = 0.012). For the cortical volume of the right fusiform gyrus, we found a significant relationship with the MMSE-K (standardized *β* = 0.2601, *p* = 0.017), CERAD-K CR (standardized *β* = 0.2546, *p* = 0.011), and total scores (standardized *β* = 0.1881, *p* = 0.042) in the Aβ − group, adjusted for age, sex, years of education, total intracranial volume, and *APOE* ε4 carrier status (Figure 2).

In the *APOE* ε4 non-carrier group, there was a significant association between CERAD-K CR/total scores and cortical volume of the left fusiform gyrus, after adjusting for age, sex, years of education, total intracranial volume, and Aβ deposition (Figure 2, CERAD-K CR, standardized *β* = 0.2955, *p* = 0.001; CERAD-K Total, standardized *β* = 0.1885, *p* = 0.042).

## Discussion

The current study aimed to investigate whether the association between years of education and cortical volume in AD-vulnerable brain regions is moderated by AD risk factors, including Aβ deposition and *APOE* ε4 carrier status, in the preclinical phase. In this study, the volume of the left lateral occipital cortex and bilateral fusiform gyrus showed a significant differential association with years of education, depending on the presence of AD risk factors. In addition, a significant association between bilateral fusiform gyrus cortical volume and AD-nonspecific cognitive function was observed, predominantly in individuals without AD risk factors.

With respect to the main objective of this study, we found a significant education year-by-Aβ deposition interaction for cortical volume in the left lateral occipital cortex and right fusiform gyrus. This result contributed to Aβ + cognitively normal older adults showing higher education years with lower cortical volume in specific AD-vulnerable brain regions. Higher years of education may have contributed to higher cognitive reserve, allowing cognitive function to remain within the normal range, even when the main pathology of AD has been deposited. However, given the deteriorating effect of Aβ deposition on cortical volume (Becker et al., 2011), this intermediate phenotype may have decreased because the threshold for tolerating Aβ deposition increased with higher cognitive reserve (Rentz et al., 2010). Regarding the brain regions that showed significant interactions in this study, the cortical thickness of the lateral occipital cortex displayed a significant interaction with cognitive reserve status for fluid reasoning in CN older adults (Stern et al., 2018). Furthermore, the lateral occipital cortex has been identified as a region that is activated as a form of neural compensation for increased cognitive loading in older adults with Aβ deposition (Elman et al., 2014). However, we could only identify a significant association between left lateral occipital cortex volume and CP scores among individuals who were Aβ + CN. Although visuospatial function is not a type of cognitive function sensitive to AD (Karrasch et al., 2005), several key previous studies have reported a relationship between the lateral occipital complex and visuospatial function (Grill-Spector et al., 2001; James et al., 2003; Dilks et al., 2011; Pascoal et al., 2020). Therefore, the results of this study should be cautiously interpreted. It is also worth noting that the lateral occipital cortex corresponds to Braak stage V and that tau deposition in this region is present in a smaller proportion among individuals who were Aβ + CN (Pascoal et al., 2020). Additionally, prior research has shown that Aβ deposits in the preclinical phase are the strongest predictors of tau accumulation (Ossenkoppele et al., 2021), which has been demonstrated to affect cortical atrophy in the course of AD (Harrison et al., 2019). Considering the lack of a mediating effect of Aβ accumulation between years of education and cortical volume in the present study, it may be that older adults with higher years of education who exhibit Aβ + CN are more likely to be susceptible to advanced tau deposition, which may cause atrophy in related brain regions. In addition, we found no significant association between the years of education and cortical volume in AD-vulnerable brain regions among cognitively normal older adults. However, in participants with AD risk factors, we observed a significant negative association between years of education and cortical volume with the education years-by-AD risk factor interaction. It might be that this interaction was primarily observed in regions with relatively advanced Braak stages, where the effect of years of education on increasing thresholds for AD pathologies such as tauopathy may be more prominent. Furthermore, cognitive reserve factors such as years of education also influence brain maintenance, which is the ability to prevent brain changes associated with AD pathology (Stern et al., 2023). Therefore, differences in brain maintenance in each brain region may have affected the brain regions that displayed significant interactions. In this regard, by longitudinally measuring the intensity of atrophy in AD susceptible brain regions against Aβ and tau deposition and assessing how this affects the way cognitive function changes, we can clarify the current findings with a more comprehensive understanding of cognitive reserve and brain maintenance.

The right fusiform gyrus was another brain region that displayed a significant education year-by-Aβ deposition interaction and this region also showed a similar interaction pattern with the left lateral occipital cortex. Furthermore, the accumulation of Aβ did not play a mediating role in the relationship between years of education and the cortical volume of the fusiform gyrus. In the previous papers, the fusiform gyrus has performed the compensatory role, interacting with the level of education in the CN and prodromal AD group (Morbelli et al., 2013). Additionally, memory training has been demonstrated to increase the cortical thickness of the right fusiform gyrus in the CN participants (Belleville and Bherer, 2012). Moreover, the fusiform gyrus is a Braak stage III brain region, and in CN individuals with Aβ deposition, tau protein deposition in this region has been reported to occur in close to 10% of cases (Pascoal et al., 2020). Building on the discussion of the lateral occipital cortex, it might be reasonable to speculate that preclinical subjects with Aβ protein may exhibit atrophy of the fusiform gyrus by advanced tau accumulation as they accrue higher years of education. In the present study, the association between cortical volume in the right fusiform gyrus and AD-prone cognitive function was not confirmed, and a significant association was only displayed in the group without concomitant Aβ deposition. In addition, despite the interaction between Aβ deposition and left fusiform gyrus cortical volume for verbal memory recognition, there was no significant association between cortical volume and this cognitive domain in each Aβ + and Aβ- group. Since the participants in the present study were recruited prior to the onset of the clinical phenotype of AD, it is improbable that clear cognitive changes related to AD were noticeable. Further longitudinal observations are also necessary to elucidate the clinical implications of alterations in the volume of the regions of interest, conjoining with the years of education.

We also identified a significant education year-by-*APOE* ε4 carrier status interaction for the cortical volume in left lateral occipital cortex and fusiform gyrus. Based on this significant finding, *APOE* ε4 carriers with higher years of education displayed lower cortical volume in regions of interest. Additionally, we discovered significant interactions in similar regions as when we utilized Aβ deposition as an effect modifier. These results need to be interpreted with caution considering that Aβ deposition occurs at an earlier age and at a faster rate in *APOE* ε4 carriers (Jack et al., 2015; Jansen et al., 2015). In the current study, the proportion of *APOE* ε4 carriers in the Aβ + group was more than twice as high as in the Aβ − group. In this regard, it is conceivable that the effect of higher years of education on raising the threshold for AD pathology is more pronounced in the *APOE* ε4 carriers. In addition, it can be assumed that intermediate phenotypes, such as cortical volume, would have been exposed to the debilitating effect of more accumulated pathology. Despite this testable prediction, in a prior study on individuals from normal cognition to MCI, the *APOE* ε4 carrier status did not influence hippocampal volume in those with high or low levels of education, respectively (Vemuri et al., 2016). However, this previous study did not evaluate effects on brain regions other than the hippocampus, and the current study also did not find significant effects on the hippocampus. Additionally, in the current findings on the *APOE* ε4 carriers, the Aβ deposition also did not play a mediating role in the relationship between years of education and the cortical volume in regions of interest. Considering the limited research available on the association between years of education, cortical volume, cognitive function, and effect modifiers such as *APOE* ε4 carrier status during the preclinical phase, further longitudinal studies are needed that comprehensively analyze the impact of cognitive reserve proxy for the relationship between atrophy of AD-prone brain regions and cognitive change, together with Aβ and tau deposition. The present study could serve as a starting point for these investigations.

While there is some evidence suggesting that the lateral occipital cortex and fusiform gyrus may have a compensatory role (Grill-Spector et al., 2001; Morbelli et al., 2013), interacting with the education years, the current study found that cortical volume was positively associated with cognitive function only in individuals who do not carry the *APOE* ε4 allele. Additionally, the present study found associations with the total cognitive function and visuospatial memory domain scores. These findings are supported by the fact that regions of interest are related to visuospatial recognition (Grill-Spector et al., 2001). However, further research is needed to determine the longitudinal effects of the cortical volume of brain regions that have education years-by-*APOE* ε4 carrier status interaction on AD-vulnerable cognitive functions.

This study has several limitations. The relatively small sample size in this study may have compromised its statistical robustness. Moreover, an imbalanced sample size might affect the external validity of the findings, making them less generalizable to the broader population. Therefore, further research should be conducted with a larger sample size, including a larger number of subjects in the Aβ + group. Additionally, we utilized years of education as a surrogate for cognitive reserve, primarily because of its general applicability and convenience as a measure. Nonetheless, a recent study has highlighted that years of education may not necessarily moderate the influence of AD biomarkers on cognitive decline in MCI and AD patients (Bauer et al., 2020). In addition, there are other proposed proxies to more accurately gauge the accumulation of cognitive reserve throughout individual life (Leon et al., 2014; Galvin et al., 2021). Engaging in further research with these alternate proxies can pave the way for a comprehensive understanding of cognitive reserves. A notable limitation of our study is its cross-sectional design. A more precise interpretation might be achieved if components pivotal to cognitive reserve, brain reserve, and maintenance—such as brain atrophy, function, pathology, and cognitive changes—were assessed longitudinally (Stern et al., 2020, 2023). Therefore, the accumulation of longitudinal data will be instrumental in deepening our understanding of these factors that modulate the progression of AD and the interrelationships among them. Finally, we conducted this study with cognitively normal older adults who had no family history of AD. It has been reported that family history of AD is related to regional medial temporal lobe atrophy, regardless of cognitive impairment (Ganske et al., 2016). Therefore, we cannot rule out the possibility that it might have affected the outcomes of this study.

Similar to predictions of cognitive trajectories based on the cognitive reserve hypothesis (Hall et al., 2007), the present study aimed to explore the effects of years of education on intermediate phenotypes, focusing on Aβ accumulation and *APOE* ε4 carrier status as effect modifiers in the preclinical phase. We found significant education years-by-AD risk factor interactions for the cortical volume of the brain regions involved in neural compensation. Additionally, there was a significant association between cortical volume and cognitive functions nonspecific to AD, primarily in individuals without AD risk factors. This study suggests that, in the earliest stages of AD, the differential role of AD risk factors should be considered in the effect of years of education on cortical volume, a representative intermediate phenotype of AD. In addition, given the long-term impact of cognitive reserve and the importance of a comprehensive reflection of AD pathology (Jack et al., 2015, 2019), further studies exploring the long-term implications of our findings would help deepen our understanding of cognitive reserve in the preclinical phase. Finally, we delineated the associations between education years and cortical gray matter volumes, examining the potential interactions with AD risk factors within a distinct Korean cohort. Our findings provide an initial foray into understanding these complex interrelationships. However, the specificity of our cohort necessitates broader validation. The Alzheimer’s Disease Neuroimaging Initiative (ADNI) or UK biobank database (Mueller et al., 2005; Sudlow et al., 2015), recognized for its comprehensive and diverse dataset, emerges as an ideal candidate for such validation. Incorporating analyses from these cohorts can enhance the robustness of our findings and assess their applicability beyond the Korean context. It’s imperative for future research to embrace such cross-cohort investigations, emphasizing the multifaceted nature of AD and the significant impact of genetic and environmental variations.

## Data availability statement

The datasets generated or analyzed during the current study are not publicly available due to the Patient Data Management Protocol of Yeouido Saint Mary’s Hospital but are available from the corresponding author upon reasonable request.

## Ethics statement

The studies involving humans were approved by Institutional Review Board of the Catholic University of Korea. The studies were conducted in accordance with the local legislation and institutional requirements. The participants provided their written informed consent to participate in this study.

## Author contributions

H-BK: conceptualization, methodology, data curation, writing – original draft, visualization, and formal analysis. S-HK: visualization and formal analysis. YU: software and investigation. S-MW: methodology, data curation, and writing – review and editing. RK, YC, JL, and DK: methodology and data curation. HL and CL: conceptualization and supervision. DWK: conceptualization, methodology, writing – review and editing, supervision, and project administration. All authors contributed to the article and approved the submitted version.

## Footnotes

^1^https://www.jamovi.org
