# Supplementary material for: Modulation of associations between education years and cortical volume in Alzheimer’s disease vulnerable brain regions by Aβ deposition and APOE ε4 carrier status in cognitively normal older adults
Source: Front Aging Neurosci. 2023 Sep 27;15:1248531. doi: 10.3389/fnagi.2023.1248531 (PMC10565031; doi:10.3389/fnagi.2023.1248531)
Supplement: Supplementary file 1 [file Table_1.DOCX]

**Modulation of Associations Between Education Years and Cortical Volume in Alzheimer's Disease Vulnerable Brain Regions by Aβ Deposition and *APOE* ε4 Carrier Status in Cognitively Normal Older Adults**

**Hak-Bin Kim^1^, Sung-Hwan Kim^2^, Yoo Hyun Um^3^, Sheng-Min Wang^2^, Regina EY Kim^4^, Yeong Sim Choe^4^, Jiyeon Lee^4^, Donghyeon Kim^4^**, **Hyun Kook Lim^2,4^, Chang Uk Lee^1^, and Dong Woo Kang^1*^**

^1^Department of Psychiatry, Seoul St. Mary’s Hospital, College of Medicine, The Catholic University of Korea, Seoul, Republic of Korea

^2^Department of Psychiatry, Yeouido St. Mary’s Hospital, College of Medicine, The Catholic University of Korea, Seoul, Republic of Korea

^3^Department of Psychiatry, St. Vincent’s Hospital, College of Medicine, The Catholic University of Korea, Suwon, Republic of Korea

^4^Research Institute, NEUROPHET Inc.; Seoul 06247, Republic of Korea

***Correspondence:** Dong Woo Kang, MD, PhD

Department of Psychiatry, Seoul St. Mary’s Hospital, College of Medicine, The Catholic University of Korea, 222, Banpo-daero, Seocho-gu, Seoul, 06591, Republic of Korea

Tel: +82-2-2258-6025, Fax: +82-2-536-8744, E-mail: [kato7@hanmail.net](mailto:kato7@hanmail.net)

**Running title: education years and cortical volume**

# Supplementary Methods

## Neuropsychological evaluation

# Cognitive status was assessed using neuropsychological testing at Yeouido St. Mary’s Hospital, The Catholic University of Korea. Cognitive function in all subjects was assessed using the Korean version of the Consortium to Establish a Registry for Alzheimer’s Disease (CERAD-K), which includes verbal fluency (VF), the 15-item Boston Naming Test (BNT), MMSE-K, Word List Memory (WLM), Word List Recall (WLR), Word List Recognition (WLRc), Constructional Praxis (CP), and Constructional Recall (CR). CERAD is a standardized clinical and neuropsychological assessment battery for the evaluation of patients with AD. A neuropsychologist reviewed the results to determine whether there was evidence of cognitive impairment.

# The VF score is the number of animal names that the subject could name in one minute. The BNT scores ranged from 0 to 15 points. The MMSE-K score ranged from 0 to 30 points. The WLM scores range from 0 to 30 points. The WLR scores ranged from 0 to 10 points. The WLR scores ranged from 0 to 10 points. The WLRc scores ranged from 0 to 10 points. The CP scores ranged from 0 to 11 points. The CR scores ranged from 0 to 11 points. The total memory domain scores ranged from 0 to 67 points. Finally, the total CERAD-K score ranged from 0 to 100 points.

# To evaluate executive function, we performed the Stroop task, which requires pre-directed reactions while suppressing the dominant response, such as letter reading and color reading. The Korean Color Word Stroop Test (K-CWST) was used in the current study. The subjects were requested to read the color of letters when they were written in red, blue, yellow, and black colors within a limited time (Byeon et al., 2017). Additionally, participants carried out trail-making test B, which alternatively measured the time required to connect letters and numbers in the sequence. Finally, in the VF test, the subdomain of the CERAD-K battery counts the number of animals recalled within a minute.

## *APOE* genotyping

DNA was isolated from the blood using the QIAmp Blood DNA Maxi Kit (Qiagen, Valencia, CA, USA). Genotypes for two APOE SNPs, rs429358 (E*4) and rs7412 (E*2), were determined using TaqMan SNP genotyping assays (Applied Biosystems, Foster City, California USA,).

## PET scanners

Each scanner was commissioned by scanning an NEMA phantom and adjusting the reconstruction parameters to obtain a spatial resolution of ~6.5 mm. This optimization was performed before the patients were scanned, and the images received by GE were not subjected to any further post-processing regarding the spatial resolution.

## [^18^F]-flutemetamol PET image acquisition and processing

[^18^F] FMM was manufactured, and [^18^F] FMM-PET data were collected and analyzed as previously described (Thurfjell et al., 2014). Static PET scans were acquired 90–110 min after 185 MBq of FMM injection. MRI for each participant was used to co-register and define the ROIs and correct partial volume effects that arose from the expansion of the cerebrospinal spaces accompanying cerebral atrophy using a geometric transfer matrix.

## Specific criteria for uncontrolled multiple cardiovascular risk factors

**1.5.1 Uncontrolled Arterial Hypertension**

Systolic blood pressure (SBP) ≥ 140 mm Hg or Diastolic blood pressure (DBP) ≥ 90 mm Hg despite the use of antihypertensive medication (Mancia et al., 2013;Whelton, 2017).

**1.5.2 Uncontrolled Diabetes Mellitus**

HbA1c level ≥ 7% (Association, 2021).

**1.5.3 Uncontrolled Dyslipidemia**

LDL cholesterol > 100 mg/dL in high-risk patients or > 70 mg/dL in very high-risk patients despite lipid-lowering therapies (Grundy et al., 2019).

**1.5.4 Uncontrolled cardiac disease:**

Chronic symptoms such as angina, despite medical therapy (Fihn et al., 2014). Persistent symptoms or hemodynamic instability due to the arrhythmia and failure to control arrhythmia with medications or procedural interventions (Members et al., 2014;Al-Khatib et al., 2018).

## Potential effects of age on cortical volume displaying an interaction between years of education and Alzheimer’s disease risk factors for cortical volume in regions of interest

(A) Regions of interest: Left lateral occipital cortex

|  | | | | | | **95% Confidence Interval** | |
| --- | --- | --- | --- | --- | --- | --- | --- |
| **Fitted model** | **Estimate** | **SE** | **t** | **p** | **Stand. Estimate** | **Lower** | **Upper** |
| Age | -48.392 | 15.5 | -3.120 | 0.002 | -0.258 | -0.422 | -0.094 |

(B) Regions of interest: Right fusiform gyrus

|  | | | | | | **95% Confidence Interval** | |
| --- | --- | --- | --- | --- | --- | --- | --- |
| **Fitted model** | **Estimate** | **SE** | **t** | **p** | **Stand. Estimate** | **Lower** | **Upper** |
| Age | -49.577 | 12.0 | -4.140 | < .001 | -0.345 | -0.510 | -0.180 |

(C) Regions of interest: Left fusiform gyrus

|  | | | | | | **95% Confidence Interval** | |
| --- | --- | --- | --- | --- | --- | --- | --- |
| **Fitted model** | **Estimate** | **SE** | **t** | **p** | **Stand. Estimate** | **Lower** | **Upper** |
| Age | -47.655 | 12.7 | -3.757 | < .001 | -0.308 | -0.470 | -0.145 |

Regression models were adjusted for age, sex, total intracranial volume, and effect modifiers that were not included in each interaction evaluation. ﻿Estimate, ﻿unstandardized beta coefficients; Stand. Estimate, standardized beta coefficients; SE, standard error.

## Relationship between age and education years in total group and subgroups

(A) Total group

|  |  | Education years |
| --- | --- | --- |
| Age | Pearson's r | -0.017 |
|  | p-value | 0.853 |
|  | N | 121 |

(B) Positive Aβ deposition

|  |  | Education years |
| --- | --- | --- |
| Age | Pearson's r | -0.041 |
|  | p-value | 0.700 |
|  | N | 90 |

(C) Negative Aβ deposition

|  |  | Education years |
| --- | --- | --- |
| Age | Pearson's r | 0.067 |
|  | p-value | 0.720 |
|  | N | 31 |

(D) *APOE* ε4 carrier

|  |  | Education years |
| --- | --- | --- |
| Age | Pearson's r | 0.069 |
|  | p-value | 0.711 |
|  | N | 31 |

(E) *APOE* ε4 non-carrier

|  |  | Education years |
| --- | --- | --- |
| Age | Pearson's r | -0.040 |
|  | p-value | 0.707 |
|  | N | 90 |

## Relationship between education years and cortical volumes in regions of interest

|  |  | Education years |
| --- | --- | --- |
| Lt. entorhinal cortex | Pearson's r | 0.059 |
|  | p-value | 0.530 |
| Lt. hippocampus | Pearson's r | 0.125 |
|  | p-value | 0.183 |
| Lt. fusiform gyrus | Pearson's r | -0.051 |
|  | p-value | 0.585 |
| Lt. superior temporal gyrus | Pearson's r | 0.167 |
|  | p-value | 0.073 |
| Lt. middle temporal gyrus | Pearson's r | 0.147 |
|  | p-value | 0.115 |
| Lt. inferior temporal gyrus | Pearson's r | 0.121 |
|  | p-value | 0.194 |
| Lt. insula | Pearson's r | -0.016 |
|  | p-value | 0.861 |
| Lt. temporal pole | Pearson's r | -0.058 |
|  | p-value | 0.539 |
| Lt. posterior cingulate cortex | Pearson's r | -0.003 |
|  | p-value | 0.974 |
| Lt. precuneus | Pearson's r | -0.028 |
|  | p-value | 0.768 |
| Lt. lateral occipital cortex | Pearson's r | 0.063 |
|  | p-value | 0.501 |
| Rt. entorhinal cortex | Pearson's r | 0.025 |
|  | p-value | 0.787 |
| Rt. hippocampus | Pearson's r | 0.121 |
|  | p-value | 0.196 |
| Rt. fusiform gyrus | Pearson's r | 0.027 |
|  | p-value | 0.773 |
| Rt. superior temporal gyrus | Pearson's r | 0.165 |
|  | p-value | 0.077 |
| Rt. middle temporal gyrus | Pearson's r | 0.079 |
|  | p-value | 0.401 |
| Rt. inferior temporal gyrus | Pearson's r | -0.031 |
|  | p-value | 0.743 |
| Rt. insula | Pearson's r | -0.109 |
|  | p-value | 0.246 |
| Rt. temporal pole | Pearson's r | -0.045 |
|  | p-value | 0.634 |
| Rt. posterior cingulate cortex | Pearson's r | 0.002 |
|  | p-value | 0.986 |
| Rt. precuneus | Pearson's r | 0.072 |
|  | p-value | 0.440 |
| Rt. lateral occipital cortex | Pearson's r | 0.113 |
|  | p-value | 0.227 |

# Partial correlation analysis was adjusted for age, sex, total intracranial volume, *APOE* ε4 carrier status, and Aβ deposition.

# Supplementary References

Al-Khatib, S.M., Stevenson, W.G., Ackerman, M.J., Bryant, W.J., Callans, D.J., Curtis, A.B., Deal, B.J., Dickfeld, T., Field, M.E., and Fonarow, G.C. (2018). 2017 AHA/ACC/HRS guideline for management of patients with ventricular arrhythmias and the prevention of sudden cardiac death: a report of the American College of Cardiology/American Heart Association Task Force on Clinical Practice Guidelines and the Heart Rhythm Society. *Journal of the American College of Cardiology* 72**,** e91-e220.

Association, A.D. (2021). 6. Glycemic targets: standards of medical care in diabetes—2021. *Diabetes Care* 44**,** S73-S84.

Byeon, H., Jin, H., and Cho, S. (2017). Development of Parkinson's disease dementia prediction model based on verbal memory, visuospatial memory, and executive function. *Journal of Medical Imaging and Health Informatics* 7**,** 1517-1521.

Fihn, S.D., Blankenship, J.C., Alexander, K.P., Bittl, J.A., Byrne, J.G., Fletcher, B.J., Fonarow, G.C., Lange, R.A., Levine, G.N., and Maddox, T.M. (2014). 2014 ACC/AHA/AATS/PCNA/SCAI/STS focused update of the guideline for the diagnosis and management of patients with stable ischemic heart disease: a report of the American College of Cardiology/American Heart Association Task Force on Practice Guidelines, and the American Association for Thoracic Surgery, Preventive Cardiovascular Nurses Association, Society for Cardiovascular Angiography and Interventions, and Society of Thoracic Surgeons. *Circulation* 130**,** 1749-1767.

Grundy, S.M., Stone, N.J., Bailey, A.L., Beam, C., Birtcher, K.K., Blumenthal, R.S., Braun, L.T., De Ferranti, S., Faiella-Tommasino, J., and Forman, D.E. (2019). 2018 AHA/ACC/AACVPR/AAPA/ABC/ACPM/ADA/AGS/APhA/ASPC/NLA/PCNA guideline on the management of blood cholesterol: executive summary: a report of the American College of Cardiology/American Heart Association Task Force on Clinical Practice Guidelines. *Journal of the American College of Cardiology* 73**,** 3168-3209.

Mancia, G., Fagard, R., Narkiewicz, K., Redon, J., Zanchetti, A., Böhm, M., Christiaens, T., Cifkova, R., De Backer, G., and Dominiczak, A. (2013). 2013 ESH/ESC Guidelines for the management of arterial hypertension. *Arterial Hypertension* 17**,** 69-168.

Members, W.C., January, C.T., Wann, L.S., Alpert, J.S., Calkins, H., Cleveland Jr, J.C., Cigarroa, J.E., Conti, J.B., Ellinor, P.T., and Ezekowitz, M.D. (2014). 2014 AHA/ACC/HRS guideline for the management of patients with atrial fibrillation: a report of the American College of Cardiology/American Heart Association Task Force on Practice Guidelines and the Heart Rhythm Society. *Circulation* 130**,** e199.

Thurfjell, L., Lilja, J., Lundqvist, R., Buckley, C., Smith, A., Vandenberghe, R., and Sherwin, P. (2014). Automated quantification of 18F-flutemetamol PET activity for categorizing scans as negative or positive for brain amyloid: concordance with visual image reads. *Journal of Nuclear Medicine* 55**,** 1623-1628.

Whelton, W. (2017). 2017 Guideline for the prevention, detection, evaluation, and management of high blood pressure in adults. *J Am Coll Cardiol*.
